# Changes in Ocular Blood Flow after Ranibizumab Intravitreal Injection for Diabetic Macular Edema Measured Using Laser Speckle Flowgraphy

**DOI:** 10.1155/2020/9496242

**Published:** 2020-02-10

**Authors:** Lisa Toto, Federica Evangelista, Pasquale Viggiano, Emanuele Erroi, Giada D'Onofrio, Daniele Libertini, Annamaria Porreca, Rossella D'Aloisio, Parravano Mariacristina, Luca Di Antonio, Marta Di Nicola, Rodolfo Mastropasqua

**Affiliations:** ^1^Ophthalmology Clinic, Department of Medicine and Science of Ageing, University “G. d'Annunzio” Chieti-Pescara, Chieti 66100, Italy; ^2^Department of Economic Studies, University “G. d'Annunzio” Chieti-Pescara, Chieti 66100, Italy; ^3^IRCCS—Fondazione Bietti, Rome, Italy; ^4^Department of Medical, Oral and Biotechnological Sciences, Laboratory of Biostatistics, University “G. d'Annunzio” Chieti-Pescara, Chieti 66100, Via dei Vestini 31, Italy; ^5^Institute of Ophthalmology, University of Modena and Reggio Emilia, Modena, Italy

## Abstract

**Purpose:**

To evaluate the effects of intravitreal ranibizumab (IVR) treatment on the blood flow of the optic nerve head (ONH) and of retinal vessels of the peripapillary region of eyes with diabetic macular edema (DME) assessed using laser speckle flowgraphy (LSFG).

**Methods:**

Forty eyes of 30 patients treated with IVR for DME were included in this prospective clinical study. Mean blur rate (MBR) and relative flow volume (RFV) of the ONH and of a superior retinal artery and an inferior retinal vein of the peripapillary region were measured using LSFG at baseline, 2 weeks (T1), and 1 month (T2) after IVR injection. In addition, best-corrected visual acuity (BCVA) and central retinal thickness (CRT) were measured in all cases.

**Results:**

The BCVA improved and CRT decreased significantly during the follow-up period (*p* < 0.010). MBR-related parameters of the ONH such as MBR of all area (MA), MBR of vascular area (MV), and MBR of tissue area (MT) decreased significantly at 2 weeks after IVR compared to baseline values (MA, *p* < 0.010). MBR-related parameters of the ONH such as MBR of all area (MA), MBR of vascular area (MV), and MBR of tissue area (MT) decreased significantly at 2 weeks after IVR compared to baseline values (MA, *p* < 0.010). MBR-related parameters of the ONH such as MBR of all area (MA), MBR of vascular area (MV), and MBR of tissue area (MT) decreased significantly at 2 weeks after IVR compared to baseline values (MA, *p* < 0.010). MBR-related parameters of the ONH such as MBR of all area (MA), MBR of vascular area (MV), and MBR of tissue area (MT) decreased significantly at 2 weeks after IVR compared to baseline values (MA, *p* < 0.010). MBR-related parameters of the ONH such as MBR of all area (MA), MBR of vascular area (MV), and MBR of tissue area (MT) decreased significantly at 2 weeks after IVR compared to baseline values (MA, *p* < 0.010). MBR-related parameters of the ONH such as MBR of all area (MA), MBR of vascular area (MV), and MBR of tissue area (MT) decreased significantly at 2 weeks after IVR compared to baseline values (MA, *p* < 0.010). MBR-related parameters of the ONH such as MBR of all area (MA), MBR of vascular area (MV), and MBR of tissue area (MT) decreased significantly at 2 weeks after IVR compared to baseline values (MA, *p* < 0.010). MBR-related parameters of the ONH such as MBR of all area (MA), MBR of vascular area (MV), and MBR of tissue area (MT) decreased significantly at 2 weeks after IVR compared to baseline values (MA, *p* < 0.010). MBR-related parameters of the ONH such as MBR of all area (MA), MBR of vascular area (MV), and MBR of tissue area (MT) decreased significantly at 2 weeks after IVR compared to baseline values (MA,

**Conclusion:**

IVR injection leads to a reduction of ocular blood flow both in the ONH and in the retinal peripapillary vessels associated with peripapillary vessel constriction. The reduction of CRT and related improvement of vision may be related to the changes in ocular blood flow.

## 1. Introduction

Diabetic retinopathy (DR) is a specific microvascular complication of diabetes, which represents the leading cause of blindness in the western countries. The prevalence of DR increases with the duration of diabetes (nearly all persons with type 1 diabetes and more than 60% of those with type 2 have some retinopathy after 20 years), hypertension, pregnancy, puberty, and cataract surgery [1].

Reduction of retinopathy progression relies on optimum control of blood glucose, blood pressure, and possibly blood lipids [2–4]. It has been demonstrated that hyperglycaemia instigates a cascade of events leading to retinal vascular endothelial dysfunction. The upregulation of vascular endothelial growth factor (VEGF) and inflammatory cytokines further leads to the breakdown of blood-retinal barrier and capillary leakage with consequent accumulation of intraretinal and subretinal fluid. Diabetic macular edema (DME), which can occur at any stage of DR, is characterized by increased vascular permeability and the deposition of hard exudates at the central retina [5].

The management of DME has changed significantly in recent years. For a long time, laser photocoagulation was the standard treatment, but it was characterized by macular scars with secondary vision loss [1]. Since the recent introduction of new pharmacological treatments such as anti-VEGF, the prognosis of DME patients has improved considerably. Intravitreal injection of anti-VEGF drugs, such as ranibizumab or aflibercept, blocks VEGF and subsequently reduces macular edema [6].

Ranibizumab is a humanized monoclonal antibody fragment that targets all isoforms of VEGFA and has been approved in many countries for DME treatment [7].

Retinal imaging has evolved considerably in recent years for the assessment of diabetic retinopathy and DME allowing the evaluation of retinal morphology such as capillary network visualization and retinal thickness assessment [8].

Of note, the recent introduction of laser speckle flowgraphy (LSFG) has helped to quantify the ocular blood flow in patients with DME [9].

This device is a fundus camera equipped with a diode laser (wavelength 830 nm) to obtain images by the interferences with red blood cells moving through vessels [10].

In this study, we evaluate the effects of intravitreal injection of ranibizumab (IVR) in patients with DME. In detail, we quantify blood flow parameters in the optic nerve head and in retinal vessels of the peripapillary region using LSFG. In addition, we describe changes in retinal thickness and in visual acuity.

## 2. Materials and Methods

### 2.1. Study Participants

Forty eyes of 30 patients with type 2 diabetes mellitus and no proliferative DR complicated by DME were enrolled in this prospective study. Eyes with mild to severe stage DR according to the simplified version of the ETDRS classification were included [2]. All the patients had treatment-naive DME. The study adhered to the tenets of the Declaration of Helsinki and was approved by our Institutional Review Board “Department of Medicine and Science of Aging, University “G. d'Annunzio” Chieti-Pescara, Italy.” Written informed consent was provided for all the patients enrolled in the study. Criteria for inclusion were as follows: (1) age >18 years; (2) best-corrected visual acuity (BCVA) greater than 0.7 logMAR in the study eye at baseline examination; (3) presence of DME; and (4) central macular thickness (CMT) >300 *μ*m as measured using the spectral-domain optical coherence tomography (SD-OCT) at the baseline examination.

The exclusion criteria were as follows: (1) any previous ocular surgery (including intravitreal injections) in the last 6 months; (2) laser treatments; (3) history of glaucoma; (4) vascular retinal diseases; and (5) medium lens opacities (according to Lens Opacities Classification System).

### 2.2. Study Protocol

All the patients with no ocular or systemic contraindications to anti-VEGF treatment signing an informed consent and candidates to IVR injections for DME were included in the study.

All subjects enrolled in the study were diagnosed assessing DR and DME using color fundus photography, fluorescein angiography (FA), and SD-OCT and were evaluated with a comprehensive ophthalmologic examination including assessment of BCVA, tonometry, slit-lamp biomicroscopy, and indirect fundus ophthalmoscopy.

BCVA was assessed using the Early Treatment Diabetic Retinopathy Study (ETDRS) chart.

In addition, all patients were tested by means of LSGF (Softcare, Fukutsu, Japan) and XR Avanti® AngioVue OCTA (Optovue Inc., Fremont, CA, USA).

## 3. Procedures

### 3.1. Laser Speckle Flowgraphy Assessment

Laser speckle flowgraphy is a noninvasive technique based on the laser speckle phenomenon that allows simultaneous assessment of blood flow in the vessels of the optic nerve head, choroid, and retina, as previously described [11].

In detail, LSFG (Softcare, Fukutsu, Japan) is a fundus camera equipped with a diode laser (wavelength, 830 nm) and a charge-coupled device sensor (750 × 360 pixels), which are used to obtain images of the pattern of speckle contrast produced by interference as laser light is scattered by red blood cells moving through vessels in the ocular fundus. Light reflected from the tissue produces a speckled pattern on the plane where the area sensor is focused and reflected light from moving erythrocytes causes blurring of the speckle pattern [12]. The same site can be measured by using the autotracking system.

The primary output parameter of LSFG is mean blur rate (MBR) and of ocular blood flow expressed in arbitrary unit (AU) derived from the scattering pattern produced when the ocular fundus is irradiated with laser light. MBR represents the velocity of the blurring in the speckle pattern that is caused by blood flow [10]. Images are acquired continuously at the rate of 30 frames/sec over a 4 sec period and then averaged to produce a composite map of ocular blood flow [12].

The analysis of ONH offers additional capabilities to analyze data within the rubber band. The software can distinguish vessels and tissue and display mean bloodstream values separately within the ellipse rubber band around the optic nerve head. In ONH MV, MT and MA are used as results of analysis:

- MV: mean of vascular area (higher MBR area) in the composite map
- MT: mean of tissue area (lower MBR area) in the composite map
- MA: mean of all areas in the composite map.

The analysis software (LSFG Analyzer, version 3.1.58; Softcare Co.) also extracts the vessel diameter as expressed in pixels as well as the relative flow volume (RFV) in AU. The vessel part is automatically discriminated from tissue parts by the shape of leveled off cross section. The index RFV in the center is the area of the vessel part which is the area of cross section subtracting tissue parts.

**Figure 1:**
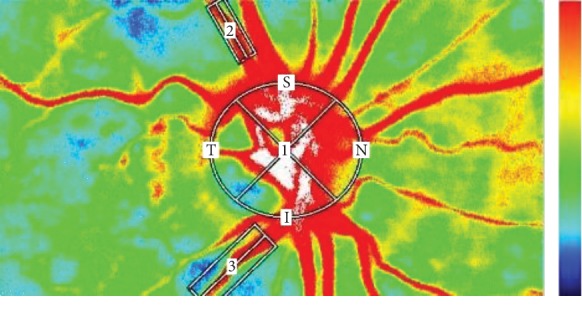
Representative composite color maps of the MBR as measured by LSFG. Red color indicates a high MBR and the blue color indicates a low MBR. To measure the MBR of the blood flow on the ONH, a circle was set around the ONH. Measurements for the retinal artery and the retinal vein were taken from sites near the ONH (within 1.5 papilla diameters). We selected in the superior region the artery and in the inferior region the vein.

All selected images were carefully visualized by two retinal specialists independently (LT and FE) to choose correctly the artery and the vein, comparing the LSFG image to a color fundus and FA images. We measured three regions: a selected retinal artery (2), a selected retinal vein (3), and the optic nerve head (ONH) (1) as shown in Figure 1. The established criteria were to select the artery in the superior region and the vein in the inferior region, in both cases from sites near the ONH (within 1.5 papilla diameters).

An elliptical rubber band is used to evaluate the flow around the optic nerve head, while a rectangle rubber band is used to read the blood flow along with a single vessel. The elliptical rubber band around the ONH was put easily on its outline, while for the rectangular ones the two retinal specialists decided to select a rectangular band that included in each case the artery and vein entirely and tissue around; thus, the rubber band diameter was variable according to the artery and vein caliber.

In addition, the LSFG Analyzer software provides other parameters characterizing the shape of the MBR waveform during one cardiac cycle for assessment of the dynamics of ocular blood flow.

Blowout time (BOT) is defined as the ratio of the half width (i.e., the time that the waveform is higher than half of the mean of the minimum and maximum signal) to the duration of one complete cardiac cycle.

Blowout score (BOS) is considered as an index of the blood flow that is maintained between heartbeats and is calculated from the difference of the maximum and the minimum MBR as well as the average MBR.

So we measured MA, MV, and MT of the ONH; MBR and RFV of retinal artery (MBR2-RFV2) and of retinal vein (MBR3-RFV3), and BOT and BOS of ONH, retinal artery, and retinal vein (BOT1, BOT2, BOT3-BOS1, BOS2, and BOS3) using the LSFG NAVI system (Softcare Co., Ltd., Fukutsu, Japan) before, 2 weeks after, and 1 month after IVR, as represented in Figure 1. The measurement conditions were kept constant as follows: angle of view, 21°; number of pixels measured, 750 × 360; and laser power, 1.37 mW. Determination of MBR was made with LSFG Analyzer software (version 3.0.47.0; Softcare Co., Fukutsu, Japan).

### 3.2. Central Macular Thickness Assessment

Central macular thickness (CMT) was manually calculated by the inbuilt caliper of the device on the macular OCT mm scan (XR Avanti®; Optovue, Inc., Fremont, CA, USA) from ILM to RPE (full retinal thickness).

## 4. Treatment

Ranibizumab 0.5 mg (0.05 mL of 10 mg/mL solution) (Lucentis; Genentech, Inc.) was injected into the vitreous cavity of all patients. All injections were performed in an operation room, and IVR was injected into the vitreous cavity through the pars plana using intravitreal injection. Patients were treated with a topical ophthalmic antibiotic for 10 days after the treatment.

### 4.1. Main Outcome Measures

**Figure 2:**
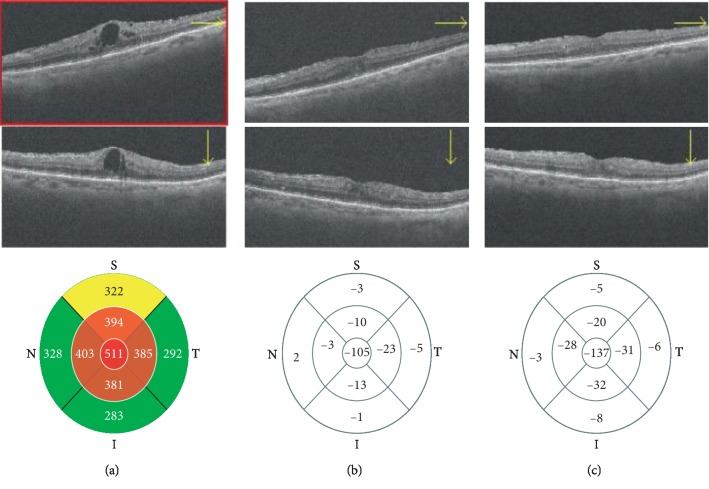
Spectral optical coherence tomography (SDOCT) images (horizontal scan, vertical scan, and thickness map) showing diabetic macular edema before (a), 15 days (b), and 30 days (c) after intravitreal injection of ranibizumab (IVR).

Best-corrected visual acuity; CMT; MA, MV, and MT of ONH; MBR and RFV of the retinal artery and vein; and BOS and BOT of ONH, retinal artery, and retinal vein were measured at baseline, 2 weeks after (T1), and 1 month (T2) after the first IVR. Representative eye with DME is shown in Figure 2.

### 4.2. Systemic Hemodynamics

The systolic blood pressure (SBP) and diastolic blood pressure (DBP) were measured at the upper arm with a sphygmomanometer before and after each examination. The mean arterial pressure (MAP) was calculated as MAP = DBP + 1/3 (SBP − DBP), and the ocular perfusion pressure (OPP) was calculated as OPP = 2/3 MAP − IOP [13].

### 4.3. Statistical Analysis

Descriptive statistics were applied to describe the sample in terms of mean and standard deviation (SD) or median and interquartile range (IQR). To determine how patients' condition improves over time, data were collected at three different time points: baseline, T1 (after 2 weeks from IVR), and T2 (after 4 weeks from IVR), respectively. Before carrying out the nonparametric analysis, normality was checked using the Shapiro–Wilks test. Because all the collected variables are not normally distributed, differences in the median values for MA, MV, MT, MBR2, MBR3, RFV2, RFV3, BOT1, BOT2, BOT3, BOS1, BOS2, BOS3, CMT, and BCVA were tested with the nonparametric Friedman's test. In addition, the Bonferroni post hoc test has been used for multiple testing. Spearman rank correlation coefficients were adopted to evaluate relations between MBR, CMT, and BCVA variables over time. For all tests, the threshold for statistical significance was set at *p*=0.05.

All analyses were performed with the open-source statistical R software (version 3.4.3, the R Foundation for Statistical Computing).

## 5. Results

Data about demographic characteristics at baseline are reported in Table 1.

There were no significant differences in SBP, DBP, and IOP, before and after the IVR, as shown in Table 2.

**Figure 3:**
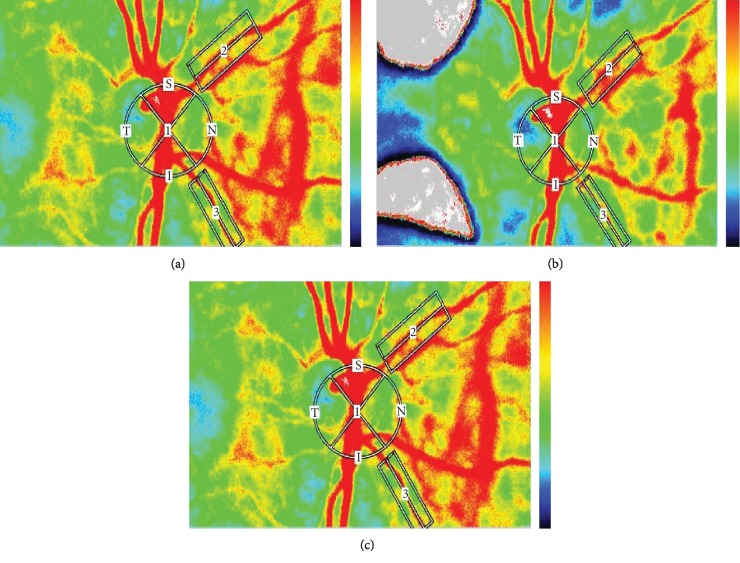
Color LSFG maps and changes of the MBR of the ONH, retinal artery, and retinal vein after intravitreal injection of ranibizumab (IVR) in a patient with diabetic macular edema in the left eye. The color LSFG map before IVR (a), 15 days after IVR (b), and 30 days after IVR (c). In the color LSFG maps, the number 1 indicates the circular scanning area for the optic nerve head, and 2 and 3 indicate the rectangular scanning areas for the retinal artery and retinal vein, respectively.

MA, MV, and MT decreased significantly at T1 compared to baseline values (MA, *p*=0.046; MT, *p*=0.023; MV, *p*=0.025), with no significant change at T2 compared to T1 (Figure 3 and Table 3). Likewise, MBR2 and MBR3 changed significantly at 2 weeks after IVR and did not change significantly thereafter (*p*=0.004 and *p*=0.010, respectively) (Figure 3 and Table 3). RFV3 also reduced significantly at T1 compared to baseline values (*p*=0.002) with no significant change at T2 compared to T1 (Table 3).

Blowout time changed significantly in ONH from 47.6 (44.2–50.2) at baseline to 54.4 (46.8–65.3) at 1 month after IVR (*p*=0.001), while it did not change significantly in the retinal artery and retinal vein (*p*=0.900, *p*=0.583, respectively). There was no statistically significant variation of BOS in both ONH and retinal peripapillary vessels (BOS 1, *p*=0.862; BOS 2, *p*=0.292; BOS 3, *p*=0.146) (Table 4).

A significant reduction of CMT was observed during follow-up (*p*=0.005) (Table 4), as shown in Figure 2.

**Figure 4:**
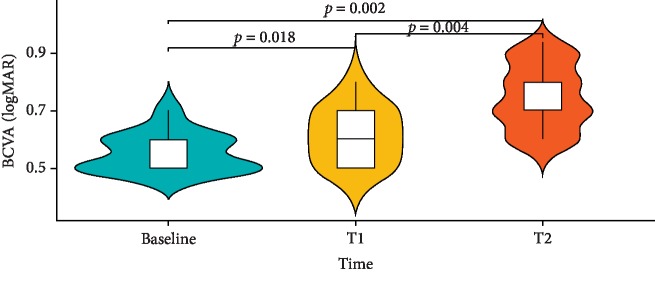
Median trend at three time points of evaluation for BCVA variable and *p* values of Bonferroni post hoc correction.

BCVA significantly improved during follow-up (*p* < 0.001) (Table 4 and Figure 4).

**Figure 5:**
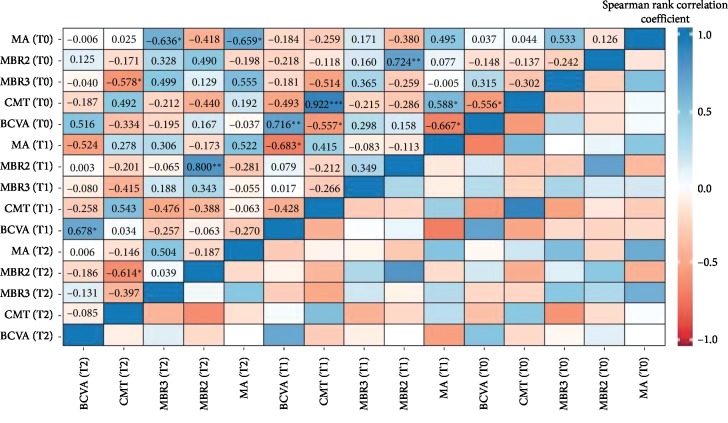
Correlations computed using Spearman rank correlation coefficient. Significance codes: ^*∗∗∗*^*p* < 0.001; ^*∗∗*^*p* < 0.01; and ^*∗*^*p* < 0.05.

A negative significant correlation coefficient was observed between CMT (T2) and MBR3 (T0) (rho = −0.578; *p*=0.05) and CMT (T2) and MBR2 (T2) (rho = −0.614; *p*=0.05). Over time, patients with low MBR values tend to have high CMT values (Figure 5).

## 6. Discussion

In this study, we investigated the effect of IVR on ocular blood flow in DR patients with DME using LSFG and we assessed central retinal thickness and visual acuity changes after treatment.

Laser speckle flowgraphy has been introduced in several ocular disorders to analyze the ocular blood flow and in detail to measure directly MBR in the retinal vessel, ONH, and choroid.

In recent years, many reports have measured retinal blood flow using LSFG in retinal diseases such as retinal vein occlusion (RVO), diabetic retinopathy, and age-related maculopathy [9, 10, 14].

Sugimoto et al. have assessed the effects of unilateral IVR on the ocular circulation of patients treated for DME and ME in RVO eyes [13]. They showed a reduction of the MBR of the optic head and of CMT after treatment in the affected treated eyes, but not the fellow untreated eyes.

Fukami et al. [12] studied the effects of IVR injection in eyes affected by ME in branch RVO and found that IVR injection leads to a transient vasoconstriction of the retinal arteries and veins and a reduction of the retinal blood flow and velocity in both occluded and not occluded quadrants.

Nitta et al. [10] compared a group of patients affected by DME to a group of patients with BRVO. In the BRVO ME group, MBR did not change significantly after intravitreal bevacizumab in ONH, retinal artery, and retinal vein; on the contrary in the DME group, MBR decreased significantly in all regions.

In our study, MBR decreased significantly in ONH, retinal artery, and retinal vein, 2 weeks and 1 month after IVR compared to baseline values (ONH, *p*=0.046; retinal artery, *p*=0.04; retinal vein, *p*=0.01). Relative flow volume reduced significantly in the retinal peripapillary vein (*p*=0.002) and BOT changed significantly in ONH (*p*=0.001) during follow-up. At the same time, CMT decreased and BCVA improved significantly (*p*=0.002 and *p*=0.004, respectively). Over time, patients with low MBR values tend to have high CMT values. Since there were no significant differences in the value of MAP, the ocular perfusion cannot influence our results on retinal circulation.

The results of our study suggest that the IVR injection caused a decrease of the retinal blood flow and velocity of the ONH vessels and peripapillary artery and vein in addition to vasoconstriction of the arteries and veins following the anti-VEGF injection with a continuous effect lasting for 30 days. Consequently, a reduction of macular edema has been observed.

These results are in agreement with data reported from other studies [12, 13] confirming the reduction of MBR of ONH in diabetic eyes after IVR and results of ocular circulation changes after IVR in other retinal diseases such as RVO describing a reduction of MBR of ONH and of RFV of retinal peripapillary vessels.

In the study of Fukami et al. [12] which is consistent with our results, the reduction of MBR and RFV was detected at 30 days after the injection. A subsequent increase of these parameters was reported at 2 months showing the temporary action of the anti-VEGF drug.

It is known that VEGF causes vasodilation of the retinal vessels with the increase in the retinal blood flow and velocity probably due to an increased production of nitric oxide.

Therefore, anti-VEGF possibly reduces retinal circulation and leads to the modification of the blood vessel caliber with vasoconstriction, and this leads consequently to ME regression [13].

In cynomolgus monkeys and rabbits, the concentration of ranibizumab in the ocular compartment (vitreous, retina, and aqueous humor) decays by 50% approximately every 3 days. After injection of 0.5 mg of ranibizumab in rabbit eyes, concentrations of >0.1 *μ*g/ml of ranibizumab were maintained in the vitreous humor for 29 days [15]. The pharmacokinetics of ranibizumab confirms the temporary changes of ocular blood flow reported in LSFG studies.

In addition, correlation analysis between vascular features at LSFG and retinal thickness allows to determine prognostic factors for ME resolution.

There are limitations in our study including the small sample size and short follow-up. Nevertheless, considering the action duration of ranibizumab as reported by pharmacokinetics studies, a follow-up of 1 month allows to discover the main changes of ocular circulation. A longer follow-up could disclose the addictive effect of repeated injections of anti-VEGF and consequent effect on ONH and retinal vessels.

In a previous study evaluating VEGF concentration in aqueous humor of diabetic eyes treated with anti-VEGF for DME, VEGF showed decreasing values after subsequent injections.

In addition, a comparison with other anti-VEGF drugs such as aflibercept, bevacizumab, or long-acting new molecules such as brolucizumab could disclose differences in ocular circulation modifications.

## 7. Conclusions

In conclusion, our results showed that an IVR injection leads to a decrease of ocular blood flow in ONH and in the peripapillary retinal vessels evaluated using LSFG that was related to a reduction of CMT and improvement of BCVA.

LSFG is confirmed to be a valuable tool for noninvasive to monitor ONH, arteriolar, and venule changes of these patients. Larger cohort studies are needed to further evaluate the potential of LSFG for the screening of DR in order to improve the treatment of diabetes-related ocular disease and to understand the relevance of intraocular early signs in the progression of the systemic disease.

## Figures and Tables

**Table 1:** Characteristics of the study group at baseline.

	Study group (*n* = 30)
Age (years), mean ± SD	65.4 ± 6.5
Gender, *n* (%)	
Male	14 (46.7)
Female	16 (53.3)
BCVA (logMAR), median (1st quartile–3rd quartile)	0.50 (0.50–0.60)
Diabetes duration (years), mean ± SD	16.4 ± 5.2
HbA1c (%), mean ± SD	8.18 ± 0.72
Insulin-dependent (yes/no)	25/5
Phakic eye, *n*	27
CMT (*μ*m), median (1st quartile–3rd quartile)	452 (352.0–514.0)

BCVA: best-corrected visual acuity; CMT: central macular thickness.

**Table 2:** Characteristics of the study group at baseline, time 1, and time 2.

Variables	Baseline	T1	T2	Friedman test
				*p* value
SBP	135 (130–140)	132 (125–140)	130 (130–135)	0.076
DBP	75 (75–80)	75 (70–75)	75 (75–77)	0.176
IOP	16 (15–17)	15.5 (15–17)	16 (15–17)	0.987
MAP	97.3 (91.5–99.8)	94.8 (89.8–96.45)	94.8 (92.3–96.5)	0.061
OPP	46.9 (44.4–50.9)	46.6 (42.3–48.8)	45.4 (44.5–47.1)	0.115

SBP: systolic blood pressure; DBP: diastolic blood pressure; IOP: intraocular pressure; MAP: mean arterial pressure; OPP: ocular perfusion pressure.

**Table 3:** Median (1st quartile–3rd quartile) computed for mean blur rate of all areas (MA), mean blur rate of vascular area (MV), mean blur rate of tissue area (MT), mean blur rate (MBR), relative flow volume (RFV) at baseline, time 1 (T1), and time 2 (T2).

Variables	Baseline	T1	Relative change	T2	Relative change	Friedman test
			(T1 vs. Baseline)		(T2 vs. T1)	*p* value
MA (AU)	25.0 (21.1–31.5)	19.7 (18.7–23.0)	−22.8 (−26.1; −6.9)^*∗*^	19.6 (18.3–26.3)	0 (−14.3; −5.1)	**0.046**
MT (AU)	14.6 (12.9–15.7)	12.0 (11.5–15.4)	−8.7 (−18.9; −4.3)^*∗*^	11.6 (11.2–15.0)	−2.3 (−2.88; −2.0)	**0.023**
MV (AU)	46.1 (40.3–55.9)	40.4 (36.5–46.5)	−13.1 (−21.2; −1.6)^*∗*^	39.7 (35.8–45.9)	−1.4 (−1.8; −1.0)	**0.025**
MBR2 (AU)	20.9 (18.0–23.7)	18.5 (15.5–20.3)	−17.1 (−26.7; −1.7)^*∗*^	16.0 (13.4–20.3)	−8.0 (−6.4; −19.7)	**0.004**
MBR3 (AU)	20.9 (20.1–25.1)	19.4 (18.4–22.1)	−6.3 (−7.9; −2.6)^*∗*^	20.6 (17.7–20.9)	−4.3 (−14.4; −10.8)	**0.010**
RFV2 (AU)	269.5 (221.6–285.5)	173.8 (126.1–206.2)	−8.9 (−44.8; −9.2)^*∗*^	199.7 (152.4–204.7)	10.8 (−19.7; −3.5)	0.292
RFV3 (AU)	306.8 (285.6–372.4)	263.2 (226.4–268.4)	−14.0 (−21.3; −7.2)^*∗*^	259.1 (140.9–336.8)	−6.1 (−19.3; −28.7)	**0.002**

^*∗*^ *p* < 0.05 nonparametric pairwise multiple comparisons versus previous evaluation time.

**Table 4:** Median (1st quartile–3rd quartile) computed for blowout score (BOS), blowout time (BOT), central macular thickness (CMT), and best-corrected visual acuity (BCVA) at baseline, time 1 (T1), and time 2 (T2).

Variables	Baseline	T1	T2	Friedman test
				*p* value
BOS1	68.7 (60.2–71.4)	63.1 (58.4–70.6)	60.5 (44.9–70.5)	0.862
BOS2	53.6 (47.3–63.5)	56.7 (50.3–57.7)	51.4 (50.9–59.5)	0.292
BOS3	65.8 (60.5–79.3)	60.3 (50.9–74.8)	72.5 (35.4–75.8)	0.146
BOT1	47.6 (44.2–50.2)	42.6 (42.0–47.6)	54.4 (46.8–65.3)^*∗*^	**0.001**
BOT2	42.7 (34.3–58.1)	38.0 (28.0–39.4)	39.5 (33.5–51.4)	0.090
BOT3	48.3 (35.9–56.3)	39.9 (29.9–46.6)	50.3 (41.3–59.1)	0.583
CMT (*μ*m)	452.0 (352.0–514.0)	400.0 (316.0–475.0)	313.0 (308.0–371.0)	**0.005**
BCVA (logMAR)	0.5 (0.50–0.60)	0.6 (0.50–0.70)^*∗*^	0.7 (0.70–0.80)^*∗*^	**<0.001**

^*∗*^ *p* < 0.05 multiple comparison test vs. previous time point.

## Data Availability

The data used to support the findings of this study are available from the corresponding author upon request.
