# Corrigendum to “Changes in Ocular Blood Flow after Ranibizumab Intravitreal Injection for Diabetic Macular Edema Measured Using Laser Speckle Flowgraphy”

**DOI:** 10.1155/2020/6219089

**Published:** 2020-07-01

**Authors:** Lisa Toto, Federica Evangelista, Pasquale Viggiano, Emanuele Erroi, Giada D'Onofrio, Daniele Libertini, Annamaria Porreca, Rossella D'Aloisio, Mariacristina Parravano, Luca Di Antonio, Marta Di Nicola, Rodolfo Mastropasqua

**Affiliations:** ^1^Ophthalmology Clinic, Department of Medicine and Science of Ageing, University “G. d'Annunzio” Chieti-Pescara, Chieti 66100, Italy; ^2^Department of Economic Studies, University “G. d'Annunzio” Chieti-Pescara, Chieti 66100, Italy; ^3^IRCCS—Fondazione Bietti, Rome, Italy; ^4^Department of Medical, Oral and Biotechnological Sciences, Laboratory of Biostatistics, University “G. d'Annunzio” Chieti-Pescara, Chieti 66100, Via dei Vestini 31, Italy; ^5^Institute of Ophthalmology, University of Modena and Reggio Emilia, Modena, Italy

In the article titled “Changes in Ocular Blood Flow after Ranibizumab Intravitreal Injection for Diabetic Macular Edema Measured Using Laser Speckle Flowgraphy” [1], there was an error in the corresponding author's name in the article. This is corrected as shown below.

Correspondence should be addressed to Federica Evangelista federica.evan@hotmail.com.

Also, the brand of the instrument used was listed incorrectly in the Materials and Methods and Procedures section; it has now been changed to NIDEK Co., LT Gamagori, Aichi, Japan. The corrected section is shown below:

## 2. Materials and Methods

### 2.2. Study Protocol

All the patients with no ocular or systemic contraindications to anti-VEGF treatment signing an informed consent and candidates to IVR injections for DME were included in the study.

All subjects enrolled in the study were diagnosed assessing DR and DME using color fundus photography, fluorescein angiography (FA), and SD-OCT and were evaluated with a comprehensive ophthalmologic examination including assessment of BCVA, tonometry, slit-lamp biomicroscopy, and indirect fundus ophthalmoscopy.

BCVA was assessed using the Early Treatment Diabetic Retinopathy Study (ETDRS) chart.

In addition, all patients were tested by means of LSGF (NIDEK Co., LT Gamagori, Aichi, Japan) and XR Avanti® AngioVue OCTA (Optovue Inc., Fremont, CA, USA).

## 3. Procedures

### 3.1. Laser Speckle Flowgraphy Assessment

Laser speckle flowgraphy is a noninvasive technique based on the laser speckle phenomenon that allows simultaneous assessment of blood flow in the vessels of the optic nerve head, choroid, and retina, as previously described [11].

In detail, LSFG RetFlow (NIDEK Co., LT Gamagori, Aichi, Japan) is a fundus camera equipped with a diode laser (wavelength, 830 nm) and a charge-coupled device sensor (750 × 360 pixels), which are used to obtain images of the pattern of speckle contrast produced by interference as laser light is scattered by red blood cells moving through vessels in the ocular fundus. Light reflected from the tissue produces a speckled pattern on the plane where the area sensor is focused, and reflected light from moving erythrocytes causes blurring of the speckle pattern [12]. The same site can be measured by using the autotracking system.

The primary output parameter of LSFG is the mean blur rate (MBR) and of ocular blood flow expressed in arbitrary unit (AU) derived from the scattering pattern produced when the ocular fundus is irradiated with laser light. MBR represents the velocity of the blurring in the speckle pattern that is caused by blood flow [10]. Images are acquired continuously at the rate of 30 frames/sec over a 4 sec period and then averaged to produce a composite map of ocular blood flow [12].

The analysis of ONH offers additional capabilities to analyze data within the rubber band. The software can distinguish vessels and tissue and display mean bloodstream values separately within the ellipse rubber band around the optic nerve head. In ONH MV, MT and MA are used as results of analysis:

- MV: mean of vascular area (higher MBR area) in the composite map
- MT: mean of tissue area (lower MBR area) in the composite map
- MA: mean of all areas in the composite map

The analysis software LSFG Analyzer (NIDEK Co., LT Gamagori, Aichi, Japan) also extracts the vessel diameter as expressed in pixels as well as the relative flow volume (RFV) in AU. The vessel part is automatically discriminated from tissue parts by the shape of leveled off cross-section. The index RFV in the center is the area of the vessel part which is the area of cross-section subtracting tissue parts.

All selected images were carefully visualized by two retinal specialists independently (LT and FE) to choose correctly the artery and the vein, comparing the LSFG RetFlow image to a color fundus and FA images. We measured three regions: a selected retinal artery (2), a selected retinal vein (3), and the optic nerve head (ONH) (1) as shown in Figure 1. The established criteria were to select the artery in the superior region and the vein in the inferior region, in both cases from sites near the ONH (within 1.5 papilla diameters).

An elliptical rubber band is used to evaluate the flow around the optic nerve head, while a rectangle rubber band is used to read the blood flow along with a single vessel. The elliptical rubber band around the ONH was put easily on its outline, while for the rectangular ones, the two retinal specialists decided to select a rectangular band that included in each case the artery and vein entirely and tissue around; thus, the rubber band diameter was variable according to the artery and vein caliber.

In addition, the analysis software LSFG Analyzer (NIDEK Co., LTD., Gamagori, Aichi, Japan) provides other parameters characterizing the shape of the MBR waveform during one cardiac cycle for assessment of the dynamics of ocular blood flow.

Blowout time (BOT) is defined as the ratio of the half-width (i.e., the time that the waveform is higher than half of the mean of the minimum and maximum signal) to the duration of one complete cardiac cycle.

Blowout score (BOS) is considered as an index of the blood flow that is maintained between heartbeats and is calculated from the difference of the maximum and the minimum MBR as well as the average MBR.

So we measured MA, MV, and MT of the ONH; MBR and RFV of the retinal artery (MBR2-RFV2) and of the retinal vein (MBR3-RFV3); and BOT and BOS of the ONH, retinal artery, and retinal vein (BOT1, BOT2, BOT3-BOS1, BOS2, and BOS3) using the LSFG NAVI system (NIDEK Co., LT Gamagori, Aichi, Japan) before, 2 weeks after, and 1 month after IVR, as represented in Figure 1. The measurement conditions were kept constant as follows: angle of view, 21°; number of pixels measured, 750 × 360; and laser power, 1.37 mW. The determination of MBR was made with to modify the analysis software LSFG Analyzer (NIDEK Co., LTD., Gamagori, Aichi, Japan).

## References

[1] Toto L., Evangelista F., Viggiano P. (2020). Changes in ocular blood flow after ranibizumab intravitreal injection for diabetic macular edema measured using laser speckle flowgraphy. *BioMed Research International*.

